# Supplementary material for: Mechanisms governing the pioneering and redistribution capabilities of the non-classical pioneer PU.1
Source: Nat Commun. 2020 Jan 21;11:402. doi: 10.1038/s41467-019-13960-2 (PMC6972792; doi:10.1038/s41467-019-13960-2)
Supplement: Supplementary file 7 — Source data [file 41467_2019_13960_MOESM7_ESM.zip › Source_Data/FigureS2/FigureS2B_MotifScanOutput/KmeansCluster7/homerResults.html]

/misc/data/analysis/project\_PU1/CTV1/motifs/basic/PU1\_Kmeans14.7/final - Homer de novo Motif Results


# Homer *de novo* Motif Results (/misc/data/analysis/project\_PU1/CTV1/motifs/basic/PU1\_Kmeans14.7/final)

Known Motif Enrichment Results  
Gene Ontology Enrichment Results  
If Homer is having trouble matching a motif to a known motif, try copy/pasting the matrix file into
STAMP  
More information on motif finding results: HOMER
| Description of Results
| Tips
  
Total target sequences = 3210  
Total background sequences = 43705  
\* - possible false positive  

|  |  |  |  |  |  |  |  |  |
| --- | --- | --- | --- | --- | --- | --- | --- | --- |
| Rank | Motif | P-value | log P-pvalue | % of Targets | % of Background | STD(Bg STD) | Best Match/Details | Motif File |
| 1 | C G T A C G T A C G T A T C A G T C G A T C A G C A T G C G T A C G T A T A C G G A C T | 1e-1729 | -3.983e+03 | 82.83% | 10.82% | 37.9bp (77.4bp) | PB0058.1\_Sfpi1\_1/Jaspar(0.968) More Information \| Similar Motifs Found | motif file (matrix) |
| 2 | C T G A C T A G A C T G A C T G C G T A C G T A T C A G A G T C | 1e-499 | -1.151e+03 | 63.77% | 22.46% | 54.1bp (74.9bp) | GCR2(MacIsaac)/Yeast(0.861) More Information \| Similar Motifs Found | motif file (matrix) |
| 3 | T G A C G C T A A C T G G C A T C A G T A G T C G A T C G A C T A G T C C A G T A G C T A G C T A G C T | 1e-411 | -9.483e+02 | 38.16% | 8.52% | 51.4bp (70.6bp) | SpiB(ETS)/OCILY3-SPIB-ChIP-Seq(GSE56857)/Homer(0.844) More Information \| Similar Motifs Found | motif file (matrix) |
| 4 | G T A C G C T A A G T C G A C T G A C T G A T C G A T C G T A C G A T C G T A C G C A T A G C T G A C T | 1e-406 | -9.364e+02 | 36.64% | 7.85% | 50.8bp (76.6bp) | SpiB(ETS)/OCILY3-SPIB-ChIP-Seq(GSE56857)/Homer(0.792) More Information \| Similar Motifs Found | motif file (matrix) |
| 5 | G T A C A C T G G T A C T A G C A C T G C G T A C G T A | 1e-376 | -8.678e+02 | 52.21% | 18.13% | 48.6bp (73.7bp) | RDR1/MA0360.1/Jaspar(0.790) More Information \| Similar Motifs Found | motif file (matrix) |
| 6 | C G T A C G T A G T C A T C A G A C T G A T C G C T G A C G T A | 1e-194 | -4.468e+02 | 31.87% | 11.23% | 48.5bp (75.6bp) | NDD1(MacIsaac)/Yeast(0.767) More Information \| Similar Motifs Found | motif file (matrix) |
| 7 | G T A C C T G A A T G C C G A T A C G T A G T C A C G T A T C G A G T C G C A T G C A T G A C T G C A T A G C T | 1e-170 | -3.922e+02 | 14.92% | 2.76% | 41.9bp (71.1bp) | SPI1/MA0080.4/Jaspar(0.758) More Information \| Similar Motifs Found | motif file (matrix) |
| 8 | C T G A A C T G T C A G C T G A T C G A T C G A G T A C | 1e-150 | -3.461e+02 | 35.79% | 15.94% | 55.3bp (74.5bp) | Etv2(ETS)/ES-ER71-ChIP-Seq(GSE59402)/Homer(0.967)(0.820) More Information \| Similar Motifs Found | motif file (matrix) |
| 9 | C G T A T G C A C G T A A T C G C G T A C T A G A T C G C G T A C G A T A C T G A C G T | 1e-131 | -3.023e+02 | 9.97% | 1.50% | 53.6bp (69.4bp) | SPIC/MA0687.1/Jaspar(0.831) More Information \| Similar Motifs Found | motif file (matrix) |
| 10 | C T G A C G T A C G A T C G T A A C T G C T A G A T C G C G T A C G T A A T G C | 1e-109 | -2.511e+02 | 10.56% | 2.15% | 51.1bp (77.4bp) | kni/dmmpmm(Pollard)/fly(0.745) More Information \| Similar Motifs Found | motif file (matrix) |
| 11 | G C A T C G T A T C G A G A T C G A T C C G T A A T G C G C T A | 1e-86 | -1.988e+02 | 35.86% | 20.17% | 53.6bp (70.1bp) | RUNX2(Runt)/PCa-RUNX2-ChIP-Seq(GSE33889)/Homer(0.942) More Information \| Similar Motifs Found | motif file (matrix) |
| 12 | T G A C G C T A G A T C G A T C G C A T G T A C G A T C G A T C G C A T A G T C G A T C G C A T | 1e-81 | -1.873e+02 | 9.07% | 2.12% | 49.7bp (71.9bp) | AtMYB84(MYB)/Arabidopsis thaliana/AthaMap(0.800) More Information \| Similar Motifs Found | motif file (matrix) |
| 13 | C G T A A T G C A C G T A C G T A G T C A C G T A T G C | 1e-80 | -1.860e+02 | 26.79% | 13.46% | 48.1bp (74.6bp) | RNP4F(RRM)/Drosophila\_melanogaster-RNCMPT00060-PBM/HughesRNA(0.776) More Information \| Similar Motifs Found | motif file (matrix) |
| 14 | A G C T A C T G A C G T C G A T A G T C A G T C A C G T A G T C | 1e-70 | -1.630e+02 | 9.84% | 2.81% | 48.2bp (73.3bp) | SRSF9(RRM)/Homo\_sapiens-RNCMPT00074-PBM/HughesRNA(0.791) More Information \| Similar Motifs Found | motif file (matrix) |
| 15 | G A C T A G T C A G T C G A T C G C T A A T G C A C G T A G C T A T G C A G T C A C G T C A T G A T G C | 1e-67 | -1.565e+02 | 8.97% | 2.45% | 45.1bp (77.0bp) | Eip74EF/dmmpmm(Bigfoot)/fly(0.864) More Information \| Similar Motifs Found | motif file (matrix) |
| 16 | C G T A A G T C C G T A A C T G A T C G C G T A C G T A | 1e-67 | -1.550e+02 | 21.74% | 10.64% | 52.7bp (71.2bp) | EWS:FLI1-fusion(ETS)/SK\_N\_MC-EWS:FLI1-ChIP-Seq(SRA014231)/Homer(0.902) More Information \| Similar Motifs Found | motif file (matrix) |
| 17 | C G T A T C A G G C A T A C G T G A T C G A C T A G T C T C G A A G C T T C A G T C G A A C T G C T G A | 1e-64 | -1.474e+02 | 4.05% | 0.45% | 42.0bp (78.1bp) | ZBTB33(Zf)/GM12878-ZBTB33-ChIP-Seq(GSE32465)/Homer(0.720) More Information \| Similar Motifs Found | motif file (matrix) |
| 18 | C T G A C G T A G T C A C T A G C G T A T C A G A T C G C G T A C G T A C A T G C G T A C T A G A C T G | 1e-63 | -1.460e+02 | 3.96% | 0.43% | 52.5bp (90.9bp) | SpiB(ETS)/OCILY3-SPIB-ChIP-Seq(GSE56857)/Homer(0.757) More Information \| Similar Motifs Found | motif file (matrix) |
| 19 | A G T C A G T C T C G A A G C T C T A G C T G A G A C T C G T A C T A G C A G T T C G A C T G A T C A G A C G T | 1e-62 | -1.435e+02 | 4.77% | 0.71% | 62.2bp (70.7bp) | FZF1/MA0298.1/Jaspar(0.594) More Information \| Similar Motifs Found | motif file (matrix) |
| 20 | A G T C G C T A A G T C T G C A G A T C G A C T A G C T A G C T A G C T T G C A C T G A T G C A T A G C | 1e-55 | -1.278e+02 | 3.40% | 0.36% | 34.7bp (74.5bp) | AT1G69570(C2C2dof)/col-AT1G69570-DAP-Seq(GSE60143)/Homer(0.645) More Information \| Similar Motifs Found | motif file (matrix) |
| 21 | C T A G C G T A A C T G C G T A C T G A A G T C C G A T A G T C G T C A A T G C A G C T G A T C | 1e-55 | -1.274e+02 | 3.49% | 0.39% | 52.0bp (72.2bp) | STZ(C2H2)/colamp-STZ-DAP-Seq(GSE60143)/Homer(0.688) More Information \| Similar Motifs Found | motif file (matrix) |
| 22 | A C G T T C G A T C G A T C A G A G C T A C G T A G C T A G T C A G T C C G A T C T A G C T G A T A C G | 1e-51 | -1.196e+02 | 3.18% | 0.34% | 77.6bp (70.6bp) | EWS:ERG-fusion(ETS)/CADO\_ES1-EWS:ERG-ChIP-Seq(SRA014231)/Homer(0.684) More Information \| Similar Motifs Found | motif file (matrix) |
| 23 | T G A C G T C A C T A G C T G A C T G A T A G C T C G A C T G A C T A G T C A G C G T A C G T A A T C G G A C T | 1e-51 | -1.178e+02 | 3.08% | 0.32% | 37.4bp (66.1bp) | ELF5(ETS)/T47D-ELF5-ChIP-Seq(GSE30407)/Homer(0.806) More Information \| Similar Motifs Found | motif file (matrix) |
| 24 | G C A T A G C T A G C T G C T A C T G A C T G A T A G C T C G A C G T A A T G C G T A C C G T A | 1e-50 | -1.156e+02 | 2.55% | 0.19% | 40.4bp (69.2bp) | PB0093.1\_Zfp105\_1/Jaspar(0.663) More Information \| Similar Motifs Found | motif file (matrix) |
| 25 | G A T C G T A C A G T C A G T C G A T C G C A T A C G T C A T G A G T C G C A T A T C G A G C T A G C T A G T C | 1e-50 | -1.151e+02 | 3.18% | 0.36% | 58.5bp (73.7bp) | Unknown-ESC-element(?)/mES-Nanog-ChIP-Seq(GSE11724)/Homer(0.695) More Information \| Similar Motifs Found | motif file (matrix) |
| 26 | A G T C G T C A C G T A A G T C A G T C C G T A A C T G C G T A A C G T A G T C A C G T | 1e-49 | -1.150e+02 | 3.89% | 0.60% | 35.0bp (67.7bp) | GATA14(C2C2gata)/col-GATA14-DAP-Seq(GSE60143)/Homer(0.846) More Information \| Similar Motifs Found | motif file (matrix) |
| 27 | A G C T T G A C G C A T A G T C T C A G C G A T C T A G T G C A G A C T C G T A T A C G G A C T C T A G | 1e-49 | -1.146e+02 | 2.99% | 0.31% | 51.0bp (65.8bp) | FZF1/MA0298.1/Jaspar(0.718) More Information \| Similar Motifs Found | motif file (matrix) |
| 28 | A C T G C G T A C G T A A C T G C G T A C G T A A T C G | 1e-49 | -1.141e+02 | 24.36% | 14.04% | 55.4bp (84.4bp) | TRA2(RRM)/Drosophila\_melanogaster-RNCMPT00078-PBM/HughesRNA(0.952) More Information \| Similar Motifs Found | motif file (matrix) |
| 29 | G A T C G C A T C G A T A G T C G A T C C G A T T A G C G A C T A G C T G T A C G A C T A C T G G T A C | 1e-44 | -1.031e+02 | 4.30% | 0.86% | 57.1bp (71.2bp) | SPIC/MA0687.1/Jaspar(0.673) More Information \| Similar Motifs Found | motif file (matrix) |
| 30 | G T A C G A T C G A T C A G T C A G T C T G C A A G C T T C A G T G C A G A C T A G T C G A T C T C G A C T G A | 1e-41 | -9.565e+01 | 2.77% | 0.34% | 72.7bp (65.5bp) | Atf4(bZIP)/MEF-Atf4-ChIP-Seq(GSE35681)/Homer(0.660) More Information \| Similar Motifs Found | motif file (matrix) |
| 31 | C A T G G T A C A G C T C T G A A G T C T C G A A G T C C G T A A T G C G A C T C A G T | 1e-41 | -9.552e+01 | 2.12% | 0.16% | 31.2bp (62.6bp) | RBM24(RRM)/Homo\_sapiens-RNCMPT00184-PBM/HughesRNA(0.686) More Information \| Similar Motifs Found | motif file (matrix) |
| 32 | A C G T G T A C G A C T T C G A A T G C G A T C T G C A A C G T C A T G T C G A G A C T C G A T T A C G | 1e-40 | -9.401e+01 | 3.33% | 0.55% | 63.6bp (61.5bp) | ATHB-7/MA0954.1/Jaspar(0.723) More Information \| Similar Motifs Found | motif file (matrix) |
| 33 | C G T A G C A T G A T C G A T C C G T A T C G A C G A T G T A C C G T A A T G C A G T C A C G T G A T C | 1e-40 | -9.264e+01 | 1.81% | 0.10% | 77.4bp (69.4bp) | DUX4/MA0468.1/Jaspar(0.664) More Information \| Similar Motifs Found | motif file (matrix) |
| 34 | A G T C C G A T C A T G A T C G A C G T A C T G C A T G A C T G C G T A A C T G C A T G A C G T C T A G C G T A | 1e-39 | -9.150e+01 | 1.50% | 0.05% | 79.8bp (59.7bp) | AtMYB84(MYB)/Arabidopsis thaliana/AthaMap(0.654) More Information \| Similar Motifs Found | motif file (matrix) |
| 35 | A G C T A G C T A G T C A G T C A C G T T C A G C G T A C T A G C T A G T A G C A G T C A G C T A G T C G T A C | 1e-37 | -8.740e+01 | 3.05% | 0.49% | 77.3bp (70.6bp) | TFAP2B(var.2)/MA0812.1/Jaspar(0.629) More Information \| Similar Motifs Found | motif file (matrix) |
| 36 | A G C T T G A C C T G A A G T C A C T G A C G T G T A C A G T C A G T C C G T A G A C T | 1e-35 | -8.239e+01 | 3.15% | 0.57% | 64.4bp (74.7bp) | hkb/MA0450.1/Jaspar(0.693) More Information \| Similar Motifs Found | motif file (matrix) |
| 37 | A G C T A T C G G T C A A T G C C G A T G A T C C T G A A G C T A G C T C G T A | 1e-35 | -8.120e+01 | 7.69% | 2.99% | 54.8bp (67.3bp) | PB0142.1\_Jundm2\_2/Jaspar(0.887) More Information \| Similar Motifs Found | motif file (matrix) |
| 38 | A G C T A G T C C G T A A C G T A C T G C T A G A C T G C T A G A T C G G C T A C T G A | 1e-33 | -7.690e+01 | 5.48% | 1.77% | 64.3bp (69.2bp) | Tv\_0259(RRM)/Trichomonas\_vaginalis-RNCMPT00259-PBM/HughesRNA(0.804) More Information \| Similar Motifs Found | motif file (matrix) |
| 39 | C A T G C T G A A C G T C G T A C T A G A C G T T A C G T C G A C T A G A C G T A C T G C G T A | 1e-32 | -7.579e+01 | 1.62% | 0.11% | 52.4bp (76.8bp) | z/dmmpmm(Down)/fly(0.662) More Information \| Similar Motifs Found | motif file (matrix) |
| 40 | C G A T T C A G G T A C A C G T G T A C C G A T C T G A C T A G C G A T C T A G C G T A | 1e-32 | -7.573e+01 | 6.29% | 2.25% | 60.1bp (64.9bp) | zen/MA0256.1/Jaspar(0.660) More Information \| Similar Motifs Found | motif file (matrix) |
| 41 | A C T G C T G A T C G A A T C G C G T A C T A G C T A G C G T A C T A G C T A G G T C A C G T A T A C G C G T A | 1e-31 | -7.344e+01 | 3.40% | 0.76% | 46.0bp (63.6bp) | TRA2(RRM)/Drosophila\_melanogaster-RNCMPT00078-PBM/HughesRNA(0.633) More Information \| Similar Motifs Found | motif file (matrix) |
| 42 | C G A T A G T C A G C T C T A G A G T C A G T C C T G A | 1e-30 | -7.032e+01 | 30.78% | 21.64% | 57.5bp (66.2bp) | NF1-halfsite(CTF)/LNCaP-NF1-ChIP-Seq(Unpublished)/Homer(0.802) More Information \| Similar Motifs Found | motif file (matrix) |
| 43 | A C T G G A T C C G A T T A C G G C A T A G C T A G T C G A C T A G T C C T A G A G C T | 1e-29 | -6.786e+01 | 2.46% | 0.42% | 52.9bp (75.6bp) | Ot\_0263(RRM)/Ostreococcus\_tauri-RNCMPT00263-PBM/HughesRNA(0.745) More Information \| Similar Motifs Found | motif file (matrix) |
| 44 | C T A G C G A T A T C G C T G A A C G T C G T A C G T A | 1e-26 | -6.191e+01 | 18.63% | 11.74% | 57.8bp (67.8bp) | TFAM/dmmpmm(Bergman)/fly(0.863) More Information \| Similar Motifs Found | motif file (matrix) |
| 45 | C A G T A G T C C G T A A G T C C T A G A C G T A T C G A C T G | 1e-26 | -6.064e+01 | 10.81% | 5.69% | 54.3bp (67.8bp) | Mycn/MA0104.3/Jaspar(0.951) More Information \| Similar Motifs Found | motif file (matrix) |
| 46 | A C G T A G T C C T G A A G C T A C T G C G T A A C G T C G T A | 1e-25 | -5.868e+01 | 8.79% | 4.30% | 52.7bp (71.5bp) | So/dmmpmm(Noyes\_hd)/fly(0.804) More Information \| Similar Motifs Found | motif file (matrix) |
| 47 | C T A G G A T C G A T C C G T A A C G T A T C G A C G T T C A G T G C A C G T A G T C A | 1e-25 | -5.792e+01 | 7.45% | 3.40% | 57.2bp (66.3bp) | INO4/INO4\_YPD/4-INO4,37-INO2(Harbison)/Yeast(0.818) More Information \| Similar Motifs Found | motif file (matrix) |
| 48 | A C G T A G T C G C T A G T A C C G T A A C G T C T A G C T A G A C G T C G T A A C G T A G T C A G C T A C T G | 1e-25 | -5.780e+01 | 1.43% | 0.14% | 54.9bp (84.8bp) | GATA14(C2C2gata)/col-GATA14-DAP-Seq(GSE60143)/Homer(0.672) More Information \| Similar Motifs Found | motif file (matrix) |
| 49 | T A C G A T G C G T C A G C A T C T A G C A T G C T A G A C T G C T A G G T C A C G T A T G C A G A T C | 1e-24 | -5.751e+01 | 1.65% | 0.21% | 62.0bp (52.0bp) | RO3G\_00049(RRM)/Rhizopus\_oryzae-RNCMPT00205-PBM/HughesRNA(0.688) More Information \| Similar Motifs Found | motif file (matrix) |
| 50 | A C G T A G T C G A C T G T A C A C G T G A T C A G C T A G T C A G T C A C G T T A C G A G T C A G C T A T C G | 1e-22 | -5.125e+01 | 0.84% | 0.03% | 31.7bp (51.8bp) | BPC1(BBRBPC)/colamp-BPC1-DAP-Seq(GSE60143)/Homer(0.764) More Information \| Similar Motifs Found | motif file (matrix) |
| 51 | A C T G A G C T C A T G A C G T T C A G A C T G G A T C | 1e-21 | -5.065e+01 | 8.82% | 4.59% | 48.9bp (69.2bp) | MET31(MacIsaac)/Yeast(0.898) More Information \| Similar Motifs Found | motif file (matrix) |
| 52 | A C T G C G T A C G T A A G T C C G A T A C T G A G T C C G T A | 1e-21 | -5.006e+01 | 5.39% | 2.25% | 59.5bp (73.0bp) | ZNF189(Zf)/HEK293-ZNF189.GFP-ChIP-Seq(GSE58341)/Homer(0.752) More Information \| Similar Motifs Found | motif file (matrix) |
| 53 | C G T A A G T C G T A C A C G T A G T C A G T C A G T C A G T C | 1e-21 | -4.933e+01 | 11.31% | 6.51% | 55.4bp (73.5bp) | PCBP2(KH)/Homo\_sapiens-RNCMPT00044-PBM/HughesRNA(0.805) More Information \| Similar Motifs Found | motif file (matrix) |
| 54 | A G T C C T G A A C T G G C T A A C T G A C T G C T G A G T C A A T C G T A C G A T G C | 1e-21 | -4.865e+01 | 6.98% | 3.35% | 53.8bp (74.8bp) | PCBP2(KH)/Homo\_sapiens-RNCMPT00044-PBM/HughesRNA(0.800) More Information \| Similar Motifs Found | motif file (matrix) |
| 55 | A C T G A T C G A T C G A T G C A G T C A C G T C A T G A T C G A C G T A C T G C T A G A C T G | 1e-20 | -4.749e+01 | 1.03% | 0.08% | 70.2bp (47.2bp) | ZNF143\|STAF(Zf)/CUTLL-ZNF143-ChIP-Seq(GSE29600)/Homer(0.692) More Information \| Similar Motifs Found | motif file (matrix) |
| 56 | A C G T A G T C A T G C A C G T A T G C A T G C A C G T A G T C | 1e-20 | -4.742e+01 | 9.94% | 5.55% | 53.1bp (82.6bp) | SRSF1(RRM)/Homo\_sapiens-RNCMPT00107-PBM/HughesRNA(0.754) More Information \| Similar Motifs Found | motif file (matrix) |
| 57 | C A T G C T A G A C T G A C T G C G T A C T A G C T A G T A G C A G T C A C G T A G T C | 1e-17 | -4.082e+01 | 2.02% | 0.49% | 71.7bp (62.1bp) | ZFX(Zf)/mES-Zfx-ChIP-Seq(GSE11431)/Homer(0.706) More Information \| Similar Motifs Found | motif file (matrix) |
| 58 | A C G T A G T C A C G T A G T C A C T G C G A T C A T G C T G A A C G T | 1e-16 | -3.912e+01 | 1.96% | 0.48% | 49.5bp (71.3bp) | SREBF2(var.2)/MA0828.1/Jaspar(0.757) More Information \| Similar Motifs Found | motif file (matrix) |
| 59 | A C T G A G T C G C T A T A G C A G T C G A C T G C A T A T G C A G C T A G C T A T G C G T C A | 1e-16 | -3.885e+01 | 0.93% | 0.09% | 47.7bp (68.4bp) | TRA2(RRM)/Drosophila\_melanogaster-RNCMPT00078-PBM/HughesRNA(0.722) More Information \| Similar Motifs Found | motif file (matrix) |
| 60 | T C G A A T G C A G T C G C T A C G A T T A C G A G T C A G C T A C G T A G T C A G T C A C G T C A T G G C A T | 1e-15 | -3.537e+01 | 1.21% | 0.20% | 68.8bp (59.9bp) | Ets1-distal(ETS)/CD4+-PolII-ChIP-Seq(Barski\_et\_al.)/Homer(0.764) More Information \| Similar Motifs Found | motif file (matrix) |
| 61 | C T G A T A G C G A C T A C T G C T A G A G T C C G A T A T C G C G A T | 1e-15 | -3.492e+01 | 3.52% | 1.43% | 54.7bp (70.3bp) | CRZ1(MacIsaac)/Yeast(0.686) More Information \| Similar Motifs Found | motif file (matrix) |
| 62 | T G A C G T A C G T C A A C G T A C T G G A C T T C G A C G T A C T A G C T G A G T A C T C G A A C G T C T A G | 1e-14 | -3.306e+01 | 2.09% | 0.63% | 48.3bp (68.6bp) | pros/dmmpmm(Bergman)/fly(0.686) More Information \| Similar Motifs Found | motif file (matrix) |
| 63 | A C T G A G C T G A C T G T C A G A T C A T G C G C T A G A T C A G C T T G C A | 1e-14 | -3.300e+01 | 5.11% | 2.55% | 58.4bp (67.5bp) | prd/dmmpmm(Bigfoot)/fly(0.688) More Information \| Similar Motifs Found | motif file (matrix) |
| 64 | A C G T C G A T A T G C A T G C A G C T A T G C G A C T A G C T A G T C T A G C G C A T A G T C A C G T A G T C | 1e-14 | -3.263e+01 | 1.34% | 0.27% | 48.4bp (89.6bp) | Trl/dmmpmm(SeSiMCMC)/fly(0.745) More Information \| Similar Motifs Found | motif file (matrix) |
| 65 | C A G T A C T G C T G A C T G A C T A G C T G A C G T A T C G A C T G A C T A G C T A G C T G A C T G A C T G A | 1e-13 | -3.197e+01 | 1.50% | 0.35% | 44.6bp (67.3bp) | AZF1/MA0277.1/Jaspar(0.697) More Information \| Similar Motifs Found | motif file (matrix) |
| 66 | A C G T G C T A T G A C A T C G C A T G A T C G C G T A T C G A G T A C G A T C | 1e-13 | -3.130e+01 | 1.28% | 0.26% | 52.5bp (60.5bp) | OPI1/MA0349.1/Jaspar(0.730) More Information \| Similar Motifs Found | motif file (matrix) |
| 67 | A G C T T G C A C G T A T A C G C G T A C A T G A C T G C T G A C T G A C A T G C A T G | 1e-13 | -3.042e+01 | 1.06% | 0.18% | 39.3bp (58.7bp) | Ets1-distal(ETS)/CD4+-PolII-ChIP-Seq(Barski\_et\_al.)/Homer(0.724) More Information \| Similar Motifs Found | motif file (matrix) |
| 68 | A G T C G C A T T A G C A G T C G A C T T G A C G T A C A T C G G A T C A G T C | 1e-13 | -3.023e+01 | 4.83% | 2.44% | 55.8bp (79.2bp) | SRSF1(RRM)/Homo\_sapiens-RNCMPT00163-PBM/HughesRNA(0.796) More Information \| Similar Motifs Found | motif file (matrix) |
| 69 | A G T C C G T A A C G T A C T G C G A T A T C G C G T A A C G T G A T C A T C G G T A C A C G T A C T G | 1e-12 | -2.982e+01 | 0.44% | 0.01% | 48.3bp (26.9bp) | GAT3(MacIsaac)/Yeast(0.681) More Information \| Similar Motifs Found | motif file (matrix) |
| 70 | C T G A A C T G C G T A A T C G A C G T C G T A A G T C C T G A A C G T A G C T A C G T A G T C A G T C A G T C | 1e-12 | -2.952e+01 | 0.34% | 0.00% | 52.4bp (41.0bp) | GFY(?)/Promoter/Homer(0.744) More Information \| Similar Motifs Found | motif file (matrix) |
| 71 | G T A C C G T A A C T G A C T G A C G T A C T G C T G A A G C T A T G C A C G T A G T C A C G T | 1e-12 | -2.929e+01 | 0.56% | 0.03% | 49.8bp (60.1bp) | At5g22890(C2H2)/col-At5g22890-DAP-Seq(GSE60143)/Homer(0.724) More Information \| Similar Motifs Found | motif file (matrix) |
